# Prevalence and Associated Factors of Eating Disorders Among Female Students at Jazan University, Kingdom of Saudi Arabia: A Survey Study

**DOI:** 10.7759/cureus.43291

**Published:** 2023-08-10

**Authors:** Suhaila A Ali, Mohammed S Mahfouz, Raghad A Hakami, Tahani H Altubayqi, Nirmin H Alhazmi, Nihal A Adawi, Raud M Khormi, Weam Yaqoub, Ghadah Maghfori, Manal H Mujarribi, Ibrahim M Dighriri

**Affiliations:** 1 Department of Family and Community Medicine, Jazan University, Jazan, SAU; 2 Faculty of Medicine, Jazan University, Jazan, SAU; 3 Faculty of Medicine, Jazan University, Jazan, SAU; 4 Department of Pharmacy, King Abdulaziz Specialist Hospital, Ta'if, SAU

**Keywords:** jazan, university students, anorexia nervosa, bulimia nervosa, eds, eating disorders

## Abstract

Background and objective: Concerns about the incidence of eating disorders (EDs) among university students are spreading throughout the world. In Saudi Arabia, little is known about the prevalence and associated factors of EDs among female university students. Thus, this study investigated the prevalence, common types, and potential associated factors of EDs among female students of Jazan University.

Methods: A cross-sectional survey was conducted between August 31, 2020, and November 2, 2020. The snowball technique was used to recruit female students via an electronic survey distributed in Arabic. The survey collected information about demographic characteristics, and SCOFF (Sick, Control, One, Fat, Food) and Eating Attitudes Test (EAT-26*)* scales. Cronbach's alpha for the SCOFF and EAT-26 scales was calculated to be 0.78 and 0.58, respectively, in this study.

Results: A total of 566 female students participated in the survey, with a mean age of 22.12 ± 2.93 years. The results showed that 47.9% of participants were at risk for EDs based on SCOFF scores, while 26.5% were at risk based on EAT-26 scores. The most common types of EDs were bulimia nervosa and binge eating disorder. Furthermore, the study identified several sociodemographic characteristics, including year of study (p = 0.042), college type (p = 0.004), body weight (p = 0.001), and BMI (p = 0.001), that are significantly associated with EDs. However, no significant relationships were observed between marital status (p = 0.103), age (p = 0.147), and height (p = 0.509) with SCOFF scores. Some students reported frequent binge eating, purging, or laxative/diet pill misuse.

Conclusions: The study revealed a moderate to high prevalence of risk for EDs among female university students in Jazan, Saudi Arabia, associated with higher study years, college majors, and body weight and BMI. Dangerous ED behaviors reported by some students signal an urgent need for resources to identify and support those suffering from these disorders. Targeted interventions and services may help address this critical issue on campuses and support vulnerable students in need. Continued research and public health action are needed to curb the spread of these disorders.

## Introduction

Eating disorders (EDs) are serious mental health issues affecting people of all ages, genders, and cultures [1,2]. Due to drastic changes in their eating practices, EDs are a significant cause of morbidity and death among teenage girls and young adult women [3]. The prevalence of overweight people is high in the Middle East and several Arab nations [4]. Since obesity is a major driving force, disordered eating attitudes are expected to increase in Arab populations [1,4,5]. Globally, in the general population, the prevalence of EDs is 5.7% in women and 2.2% in men [6]. This frequency rises substantially among medical students, with a recent meta-analysis finding 10.4% worldwide in EDs [7].

Female university students have a high frequency of EDs throughout Europe [8,9]. Peer pressure, living in dormitories, tight interactions, new social contacts, high academic demands, and increased life aspirations are all risk factors for EDs among students [10]. Ineffective time management is a common contributor to or symptom of student stress [11]. Food preferences can change due to biological and psychological changes brought on by stress [12,13]. Early diagnosis and treatment of ED can result in complete recovery [14,15]. Therefore, screening students for EDs using accurate measures can allow for early identification and medical and psychological management [16,17]. In response to different types of stress, males and females alter their eating behaviors differently. Females eat more under stress than men, who eat less, according to various studies [18,19]. In women, good and unhealthy weight management practices are 23% and 10%, respectively, while binge eating is 17% more often [20]. In the last two decades, EDs have increased substantially globally and occur in all ethnic, cultural, and socioeconomic categories, with young females aged 15-24 years being the most prone to anorexia nervosa [21].

The prevalence of EDs was attributed to anorexia nervosa and bulimia nervosa, particularly among young girls in high-income countries [3,22]. As low- and middle-income countries grow and experience cultural change, the prevalence of anorexia nervosa and bulimia nervosa in these nations can increase [3,23]. Anorexia nervosa is defined as dieting or not eating to the point where a person loses more than 15% of their weight [24,25]. The disorder is distinguished by a pathological dread or unreasonable perception of being overweight and obsessive weight loss practices [24,25]. Bulimia is also known as bulimia nervosa or binge ED [26]. It is characterized as consuming much food quickly to drive oneself to vomit [27]. Both conditions often manifest in early or mid-adolescence [27]. Anorexia and bulimia seem to have hereditary roots and are prevalent in some families [27]. Anorexia and bulimia can produce a variety of life-threatening problems, such as hormonal imbalances, menorrhagia, osteoporosis, and electrolyte imbalance, which can cause significant heart rate difficulties and even death [27,28]. The high frequency of EDs among teenage females requires psychiatric therapy, parental support, and education. Therefore, this study aims to define the prevalence, most common types, and possible associated factors of EDs among female students of the Jazan University.

## Materials and methods

Study design and setting

A cross-sectional survey was used for the research. The survey was distributed to students with a statement about the proposed study and a consent form. The study was carried out by sending an online survey to students at Jazan University, a public university located in the southwest of Saudi Arabia on the Red Sea. The university was established in 2006, under the supervision of the Saudi Ministry of Education. The study was approved by the Ethics Committee of Jazan University (approval number: REC-43/03/031).

Study duration and population

The study was carried out at a single point, from August 31, 2020, to November 2, 2020. It recruited undergraduate female students from Jazan University via an online survey designed in Google Forms (Google LLC, Mountain View, California, United States). All Jazan University from the first year to the final year who registered for the academic year 2020-2021 were included. Students who did not agree to participate in the study were excluded, as were women from outside the university.

Sample size

The Raosoft calculator (Raosoft Inc., Seattle, Washington, United States) was used to calculate the sample size. Based on the following assumptions: 95% confidence interval, 5% error margin, 50% anticipated response, and a total number of female students at Jazan University of around 25.000, the minimum required sample size was 379.

Data collection and study instrument

The recruitment process followed the snowball method. Data collection was via an electronic survey, prepared and designed in Arabic to suit the participants. The survey was modified from previous studies [1,29,30]. The survey was distributed through social media and consisted of the following sections: (A) Questions for demographic characteristics such as (sex, age, material state, education level); (B) Sick, Control, One, Fat, Food (SCOFF) questions; and (C) Eating Attitudes Test (EAT-26). SCOFF and EAT-26 are both very accurate and valid scales to assess the risk of EDs.

The SCOFF questionnaire is a widely used screening tool for EDs that assesses five factors: (i) self-induced vomiting, (ii) loss of control over eating; (iii) recent weight loss; (iv) body image distortion; and (v) food dominance. It comprises three yes/no questions and there is a score of one point for each "yes" response and zero points for each "no" answer. A score of ≥ 2 suggests that the subject will likely have anorexia or bulimia nervosa.

The EAT-26 assesses risk across three subscales: dieting, bulimia and food preoccupation, and oral control. The EAT-26 scale is divided into three subscales, each with 26 issues. Except for the 25th question, each item includes six answer alternatives with values ranging from 0 to 3 ("always" = 3, "nearly always" = 2, "often" = 1, "seldom" = 0, "hardly ever" = 0, and "never" = 0). Question 25 has a reversed score ("often," "almost usually," and "frequently" = 0, "rarely" = 1, "almost never" = 2, "never" = 3). The total score for the EAT-26 is the sum of the scores for each of the 26 items. A score of 20 indicates a "disordered eating attitude". EAT-26 subscale scores provide insight into the nature and severity of the risk to help guide intervention. The dieting subscale assesses restrictive dieting and weight concern.

The Arabic format of the SCOFF has a reliability value on the Cronbach alpha scale of 0.43 [31]. The EAT-26 scales' Arabic version was tested and proven reliable [32]. Cronbach's alpha for the Arabic SCOFF and EAT-26 scales was calculated to be 0.78 and 0.58 in this study, respectively.

Data presentation and analysis

The data were analyzed using IBM SPSS Statistics for Windows, Version 26.0 (2019; IBM Corp., Armonk, New York, United States). The mean and standard deviation for numerical variables and the percentage and frequency distribution for categorical variables were computed throughout the study. The chi-square test was used to analyze the data further to look for correlations, and the T-test was used to check for differences. The cut-offs for EAT-26 and SCOFF scores were 20 and 2, respectively. Based on these cut-offs, participants were classified as having EDs or not having EDs. The body mass index (BMI) was determined by dividing the total body weight by the square of the individual's height in meters. Participants were classified according to BMI: BMI ≥ 30.00 implied obesity, 25.00 ≤ BMI < 30 indicated overweight, 18.5 ≤ BMI< 25 was considered the normal range, and BMI < 18.5 was considered underweight. The cutoff for statistical significance was set at P < 0.05.

## Results

A total of 566 females completed the survey and were included in the study. Their mean age was 22.12 ± 2.93 years. The mean height and weight were 155.76 ± 6.42 cm and 55.10 ± 16.20 kg, respectively (Table 1).

**Table 1:** Mean distributions of sociodemographic continuous variables

Sociodemographic characteristics	Mean	SD
Age (year)	22.12	± 2.93
Height (cm)	155.76	± 6.42
Weight (kg)	55.10	± 16.20

A majority of participants were single (77.2%), followed by married participants (20.1%). Only 2.5% and 0.2% were divorced and widowed, respectively. In terms of year of study, the highest percentage was among the sixth year students (20.7%), followed by the fifth year (20.5%) and fourth-year students (16.6%). Students from the colleges of health represented 51.9% of the sample. The students from the colleges of science and literature were 29.0% and 19.1%, respectively. More than half of the participants were from villages (55.3%), while 44.7% lived in cities (Table 2).

**Table 2:** Frequency distributions of sociodemographic categorical variables

Sociodemographic characteristics		N	%
Marital status	Single	437	77.2
	Married	114	20.1
	Divorced	14	2.5
	Widower	1	0.2
Year of study	First year	67	11.8
	Second year	82	14.5
	Third year	90	15.9
	Fourth year	94	16.6
	Fifth year	116	20.5
	Sixth year	117	20.7
Type of college	Science and technology	164	29.0
	Arts and literature	108	19.1
	Health	294	51.9
Place of residence	City	253	44.7
	Village	313	55.3

**Figure 1:**
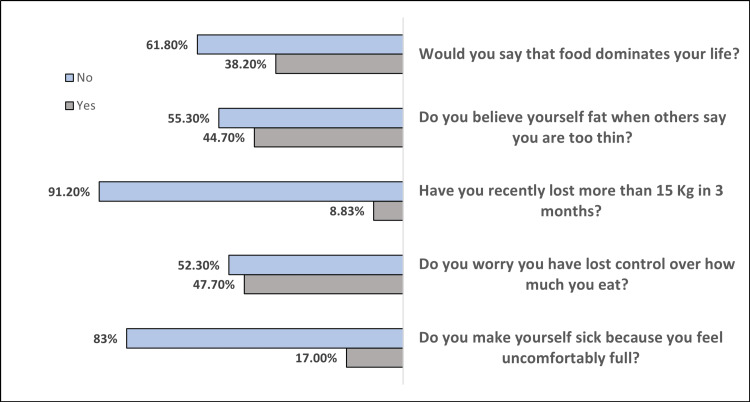
SCOFF questionnaire answered by 566 participants SCOFF: Sick, Control, One, Fat, Food

We administered the SCOFF questionnaire to 566 participants to examine ED risk. The results are shown in Figure 1. For the first item, “Do you make yourself sick because you feel uncomfortably full?”, 17% of participants responded “yes,” indicating a risk factor for bulimia. Nearly half (47.7%) reported a loss of control over eating amount (item 2), suggesting a risk for binge ED or bulimia. For item 3, “Have you recently lost more than 15 kg (33 lbs) in three months?”, 8.83% said “yes,” indicating a risk for anorexia or bulimia. Also, 44.7% reported distorted body image (item 4), and 38.20% reported that food dominates their life (item 5), suggesting a higher risk for an ED (Figure 1). The total SCOFF scores ranged from 0 to 5, with a mean of 1.56 ± 1.33, indicating that most participants reported at least one risk factor and a subset reported multiple risk factors.

**Figure 2:**
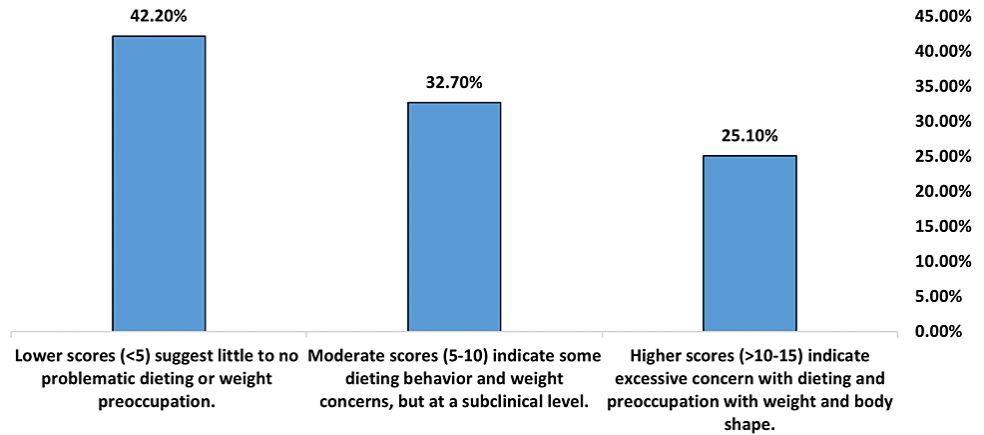
Dieting subscale (items 1-13) of EAT-26 answered by 566 participants. EAT-26: Eating Attitudes Test 26-item

The EAT-26 dieting subscale assesses restrictive dieting and weight concern. Scores averaged 7.02 (SD = 5.87). This indicates that, on average, participants reported some problems with dieting and weight, but at a level that would still be considered subclinical (Table 3). As shown in Figure 2, 25.10% of participants reported scores in the problematic range (>10-15), indicating excessive dieting behavior and preoccupation with weight and shape, and 32.70% of participants reported moderate scores (5-10), suggesting some concerns with dieting and weight that are subclinical. The largest group (42.20%) of participants reported little to no problematic dieting or weight preoccupation, with scores <5.

**Figure 3:**
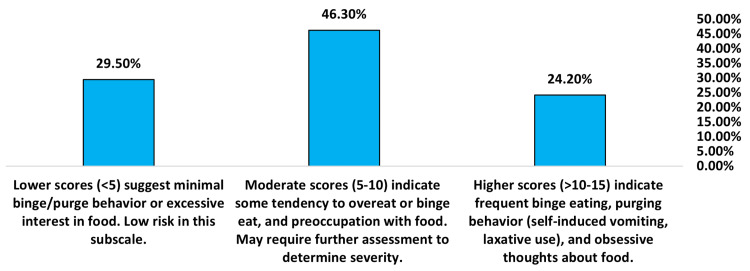
Bulimia and food preoccupation subscale (items 14-26) of EAT-26 answered by 566 participants. EAT-26: Eating Attitudes Test 26-item

The Bulimia and Food Preoccupation subscale measures thoughts about food, binge eating, and purging. Scores averaged 7.64 (SD = 5.24), indicating moderate risk overall and a need for further assessment (Table 3). As shown in Figure 3, 24.20% of participants reported scores in the problematic range (>10-15), indicating frequent binge eating, purging behavior, and obsessive thoughts about food. 46.30% of participants reported moderate scores (5-10), suggesting some tendency to overeat, binge eat, or preoccupy with food that may require further assessment. The remaining 29.50% of participants reported little to no binge/purge behavior or excessive interest in food, with scores <5 indicating a low risk.

**Table 3:** EAT-26 subscale scores for detecting EDs in 566 participants. EAT-26: Eating Attitudes Test 26-item; ED: eating disorder

EAT-26 subscale scores	Mean ± SD
Dieting subscale (items 1-13)	7.02 ± 5.87
Bulimia and Food Preoccupation subscale (items 14-26)	7.64 ± 5.24
Oral Control subscale (items 15 & 16 only)	0.68 ± 1.16
Total EAT-26 Scores	15.35 ± 10.75

**Figure 4:**
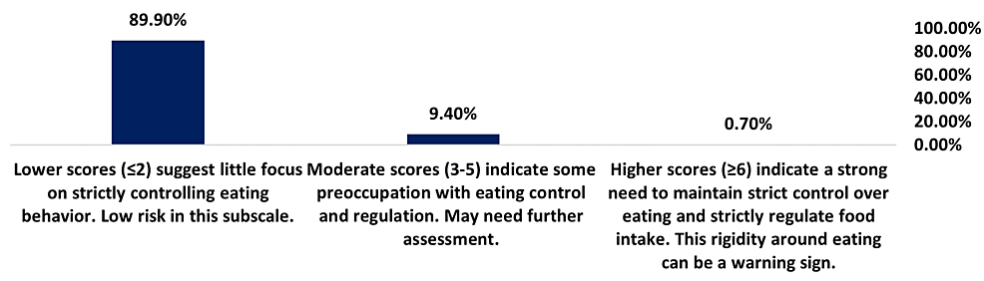
Oral control subscale (items 15 & 16 only) answered by 566 participants. EAT-26: Eating Attitudes Test 26-item

The oral control subscale assesses strict control around eating. The low average score of 0.68 (SD = 1.16) suggests a minimal problematic focus on eating control (Table 3). As shown in Figure 4, 0.70% of participants reported scores in the problematic range (≥ 6), suggesting a solid preoccupation with eating control and regulation, and 9.40% of participants reported moderate scores (3-5), indicating some focus on controlling eating that may require further assessment. The vast majority (89.90%) of participants reported little focus on strictly controlling eating behavior, with low scores (≤ 2) indicating a low risk.

Total scores on the EAT-26, where higher scores indicate greater EDs risk, averaged 15.35 (SD = 10.75). These results suggest moderate risk for EDs based on established EAT-26 score cut-offs (Table 3).

Behavioral questions suggest ED symptoms in a minority of participants. In addition to the subscale scores, participants completed three questions assessing critical behaviors associated with EDs over the past six months. Their responses, shown in Table 4, indicate that while most participants deny these harmful behaviors, a subset report symptoms at a frequency that would be considered significant. When asked how often they engage in eating binges where they feel unable to stop, 49.6% of participants said “never,” 21% said “once a month or less,” and 12.5% said “two to three times a month”. However, 4.6% reported binge eating at least two to six times a week or daily, indicating a loss of control over eating that requires assessment. When asked how often they have vomited to control their weight or shape, 83.7% of participants responded “never.” However, 5.5% reported purging through self-induced vomiting at least once a month, and 1.6% purge daily, suggesting these individuals meet the criteria for bulimia nervosa or purging disorder. When asked how often they have misused laxatives or diet pills to control their weight or shape, 84.5% of participants said “never.” But 2.3% reported misusing these methods two to six times a week, and 2.5% reported daily misuse of laxatives/diet pills for weight control. This indicates that these participants display severely unhealthy behavior and likely meet the criteria for EDs.

**Table 4:** Behavioral questions for EDs in 566 participants ED: eating disorder

Statement	N	%
In the past six months, have you gone on eating binges where you feel you may be unable to stop?		
Once a week	40	7.1
2-6 times a week	26	4.6
2-3 times a month	71	12.5
Once a month or less	119	21.0
Never	281	49.6
Once a day or more	29	5.1
In the past six months, have you ever made yourself sick (vomited) to control your weight or shape?		
Once a week	18	3.2
2-6 times a week	10	1.8
2-3 times a month	24	4.2
Once a month or less	31	5.5
Never	474	83.7
Once a day or more	9	1.6
Have you ever used laxatives or diet pills to control your weight or shape in the past six months?		
Once a week	15	2.7
2-6 times a week	13	2.3
2-3 times a month	16	2.8
Once a month or less	30	5.3
Never	478	84.5
Once a day or more	14	2.5

According to their SCOFF scores, participants were classified into those without EDs (n = 295, 52.1%) and those with EDS (n = 271, 47.9%). As shown in Table 5, there were no statistically significant differences between students with and without EDs in terms of their marital status (P = 0.103), place of living (P = 0.066), age (P = 0.147), or height (P = 0.509). However, students with EDs had significantly higher body weight and BMI than those without EDs (P = 0.001). Regarding the type of college, higher rates of EDs were among students at the colleges of science and literature compared to the colleges of health (P = 0.004). However, the year of study showed significant differences, with a higher percentage of first-year and third-year students having EDs compared to other academic years (P = 0.042). Several sociodemographic characteristics, including year of study, college type, body weight, and BMI, were significantly associated with the risk of having EDs among female university students. 

**Table 5:** Association between sociodemographic characteristics and total SCOFF score ^P^ Pearson's chi-squared test X2; ^$^ T-test; * P < 0.05 (significant) SCOFF: Sick, Control, One, Fat, Food

Sociodemographic characteristics		Without ED, N (%)	With ED, N (%)	p-value
Marital status	Single	236 (54.0%)	201 (46.0%)	0.103
	Married	49 (43.0%)	65 (57.0%)	
	Divorced	9 (64.3%)	5 (35.7%)	
	Widower	1 (100%)	0 (0%)	
	Total	295 (52.1%)	271 (47.9%)	
Year of study	First year	26 (38.8%)	41 (61.2%)	0.042*
	Second year	49 (59.8%)	33 (40.2%)	
	Third year	43 (47.8%)	47 (52.2%)	
	Fourth year	51 (54.3%)	43 (45.7%)	
	Fifth year	70 (60.3%)	46 (39.7%)	
	Sixth year	56 (47.9%)	61 (52.1%)	
Type of college	Science and technology	78 (47.6%)	86 (52.4%)	0.004*
	Arts and literature	45 (41.7%)	63 (58.3%)	
	Health	172 (58.5%)	122 (41.5%)	
Place of living	City	121 (47.8%)	132 (52.2%)	0.066
	Village	174 (55.6%)	139 (44.4%)	
BMI (kg/m^2^)	Obesity	15 (26.8%)	41 (73.2%)	0.001*
	Overweight	24 (27.3%)	64 (72.7%)	
	Normal	133 (49.4%)	136 (50.6%)	
	Underweight	123 (80.4%)	30 (19.6%)	
Age (year)		21.95 ± 2.56	22.31 ± 3.27	0.147^$^
Height (cm)		155.59 ± 6.28	155.95 ± 6.57	0.509^$^
Weight (kg)		50.66 ± 15.96	59.94 ± 15.06	0.001*^$^

Participants were classified into those without EDs (n = 416, 73.5%) and those with EDs (n = 150, 26.5%) according to their total EAT-26 scores. As depicted in Table 6, there were no statistically significant differences between students with and without EDs in terms of their marital status (P = 0.475), year of study (P = 0.315), age (P = 0.263), or height (P = 0.726). However, students with EDs had significantly higher body weight and BMI than those without EDs (P = 0.001). Significant differences were observed in the type of college and place of residence. Higher rates of EDs were found among students enrolled in colleges of science and literature and those living in cities compared to other groups (P = 0.024 and 0.004, respectively). The study found that college type, place of living, body weight, and BMI were significantly associated with the risk of having an EDs as assessed by the EAT-26 questionnaire among female university students. However, no significant relationships were observed between marital status, year of study, age, and height with EAT-26 scores. 

**Table 6:** Association between sociodemographic characteristics and total EAT-26 scores ^P^ Pearson's chi-squared test X2; $ T-test; * P < 0.05 (significant) EAT-26: Eating Attitudes Test 26-item

Sociodemographic characteristics		Without ED, N (%)	With ED, N (%)	p-value
Marital status	Single	321 (73.5%)	116 (26.5%)	0.475
	Married	86 (75.4%)	28 (24.6%)	
	Divorced	8 (57.1%)	6 (42.9%)	
	Widower	1 (100%)	0 (0%)	
	Total	416 (73.5%)	150 (26.5%)	
Year of study	First year	45 (67.2%)	22 (32.8%)	0.315
	Second year	61 (74.4%)	21 (25.6%)	
	Third year	65 (72.2%)	25 (27.8%)	
	Fourth year	73 (77.7%)	21 (22.3%)	
	Fifth year	92 (79.3%)	24 (20.7%)	
	Sixth year	80 (68.4%)	37 (31.6%)	
Type of college	Science and technology	117 (71.3%)	47 (28.7%)	0.024*
	Arts and literature	70 (64.8%)	38 (35.2%)	
	Health	229 (77.9%)	65 (22.1%)	
Place of residence	City	171 (67.6%)	82 (32.4%)	0.004*
	Village	245 (78.3%)	68 (21.7%)	
BMI (kg/m^2^)	Obesity	31 (55.4%)	25 (44.6%)	0.001*
	Overweight	48 (54.5%)	40 (45.5%)	
	Normal	210 (78.1%)	59 (21.9%)	
	Underweight	127 (83.0%)	26 (17.0%)	
Age (years), mean ± SD		22.04 ± 2.81	22.35 ± 3.23	0.263^$^
Height (cm), mean ± SD		155.70 ± 6.034	155.92 ± 7.41	0.726^$^
Weight (kg), mean ± SD		53.20 ± 15.78	60.37 ± 16.22	0.001*^$^

## Discussion

This study aimed to determine EDs' prevalence and associated factors among female students at Jazan University in Saudi Arabia. Using the SCOFF questionnaire and EAT-26 scale, the study found that the prevalence of EDs was 47.9% and 26.5%, respectively. These prevalence rates are comparable to or higher than those reported in previous studies among female university students in Saudi Arabia [1,29,33]. Previous research on the prevalence of EDs by EAT-26 scores and SCOFF scores in Palestine reported 28.6% and 38.2%, respectively [10]. The prevalence rates found in this study suggest that EDs are a significant concern among female students. This result is constant with previous research [1,29].

Several sociodemographic factors were significantly associated with increased EDs risk in our sample. Students in higher years of study and enrolled in colleges of science and literature had higher odds of being at risk of EDs based on their SCOFF scores. This may be because senior students and those in science/literature fields experience higher stress and pressure to achieve in their studies. Our findings are consistent with previous research showing a relationship between specific majors or years of study and the risk of EDs [34,35].

Students with higher body weight were also more likely to be at risk of EDs on the SCOFF and EAT-26, consistent with the literature [10,36,37]. Body weight concern and dissatisfaction are major risk factors for the development of EDs, especially in cultures that stigmatize larger body sizes [38,39]. The stigma around weight likely contributes to the moderate to high risk observed for the dieting and bulimia/food preoccupation subscales of the EAT-26 in our sample. This finding is in the same line with previous studies [39,40]. Body dissatisfaction is a risk factor for disordered eating, with higher scores linked with more severe symptoms [41]. In most cases, EDs are preceded by considerable body dissatisfaction and the adoption of weight management techniques such as restricted diets [39]. Cultural influences on body image and EDs have been widely studied, with data consistently demonstrating the impact of culture on body image and EDs [42]. Globalization and the increasing interconnectedness of societies have also shaped body image and eating disorder trends [43,44]. Body weight concern and dissatisfaction, along with cultural factors that stigmatize larger body sizes, contribute to the development of EDs. It is essential to address these risk factors and promote a more inclusive and accepting environment to help prevent the development of EDs in individuals of all body sizes.

Interestingly, students living in cities were more prone to be at risk of EDs than those from villages, according to their EAT-26 scores. This report constant with previous investigations [45-47]. This may reflect more significant body image concerns and weight stigmatization in urban compared to rural areas of Saudi Arabia. Urban living has been linked to an increased risk of EDs in previous studies [48,49]. Furthermore, rapid social change experienced by low- and middle-income countries due to globalization can also contribute to the development of EDs [50]. The shifting social norms in these regions can impact eating and weight concerns and the perception of body size ideals [45].

A subset of students in our study reported significant behavioral symptoms of EDs, including frequent binge eating, self-induced vomiting, and misuse of laxatives or diet pills. Binge eating disorder is characterized by chronic, compulsive overeating, quickly consuming large quantities of food, and feeling unable to stop eating [51]. Self-induced vomiting is an expected compensatory behavior in individuals with EDs attempting to prevent weight gain [52]. Misusing laxatives and diet pills for weight control can be dangerous. They may indicate an ED issue and need treatment and assessment [53]. These behaviors can lead to serious medical problems and even death [54]. It is vital to address these issues promptly and provide appropriate support and treatment to individuals exhibiting these behaviors. Treatment for EDs is typically an interdisciplinary approach that requires medical, nutritional, and mental treatments [55]. Early intervention is crucial to prevent complications and improve health outcomes for individuals struggling with EDs.

Study limitations

Our study has several limitations, including the cross-sectional design that precludes causality inferences. The study population was limited to students at a single university in Saudi Arabia, so findings may not generalize to other areas or groups. Further, we relied on self-reported measures of EDs rather than clinical diagnosis. However, the SCOFF and EAT-26 are well-validated tools for detecting the risk of EDs in research studies.

## Conclusions

This study found a high prevalence of risk for EDs among female students at Jazan University in Saudi Arabia based on the SCOFF questionnaire and EAT-26 scale. Nearly half of the students were at risk of EDs according to their SCOFF scores, and over a quarter were at risk based on their EAT-26 scores. Several factors were associated with increased risk, including higher years of study, enrollment in science/literature colleges, higher body weight, and urban living. These findings suggest that EDs may be an under-recognized issue affecting university women in Saudi Arabia. Some students reported engaging in dangerous ED behaviors like frequent binge eating, purging, and laxative/diet pill misuse.

University campuses may benefit from preventive interventions and early screening programs to identify students at risk for EDs. Addressing associated factors like weight stigma, body image concerns, and academic stress may help curb the development of EDs. Support services for at-risk students could include counseling and nutritional guidance. Our study adds to the limited research on EDs in Saudi Arabia and other Muslim-majority countries. We provide evidence that university women in this region may be susceptible to EDs and need resources for prevention, screening, and treatment. Longitudinal and interventional studies are warranted to understand further how to address this significant public health issue.
